# Tear Film Proteome of Healthy Domestic Cats

**DOI:** 10.1155/2021/8708023

**Published:** 2021-07-15

**Authors:** Jéssica Fontes Veloso, Paula Elisa Brandão Guedes, Luciana Carvalho Lacerda, Juliano Oliveira Santana, Irma Yuliana Mora-Ocampo, Carlos Priminho Pirovani, Arianne Pontes Oriá, Alexandre Dias Munhoz, Renata Santiago Alberto Carlos

**Affiliations:** ^1^Multidisciplinary Campus of Barra, Federal University of Western Bahia, Av. 23 de Agosto, 873, Assunção, Barra, Bahia, Brazil; ^2^Department of Agricultural and Environmental Sciences, Santa Cruz State University, Rodovia Jorge Amado, km 16, Salobrinho, Ilhéus, Bahia, Brazil; ^3^Department of Biological Sciences, Santa Cruz State University, Rodovia Jorge Amado, km 16, Salobrinho, Ilhéus, Bahia, Brazil; ^4^Department of Pathology and Clinics, Federal University of Bahia, Av. Adhemar de Barros, 500, Ondina, Salvador, Bahia, Brazil

## Abstract

The aim of this study was to investigate the proteins found in tear film of healthy domestic cats. Schirmer tear test strips were used to collect tear samples of twelve healthy cats, which were mixed, centrifuged, and placed in a single 1.5 mL microtube that was frozen at −20°C, until analysis by two-dimensional polyacrylamide gel and mass spectrometry associated with high-performance liquid chromatography. The resulting spectra were analyzed and compared with the Swiss-Prot search tool. Forty peptides were detected in the analyzed protein fragments of 90 spots, with 16 proteins identified. Of these, the authors confirmed what has been already found in other studies: lactotransferrin, serum albumin, allergenic lipocalins, and neutrophil gelatinase-associated lipocalin. Others were considered novel in tear film samples of all species: cyclin-dependent protein kinase, serine/arginine repetitive matrix protein, apelin receptor, secretory protein related to C1q/TNF, Wee1, *α*-1,4 glucan phosphorylase, and WD repeat domain 1. The network was divided into 11 clusters, and a biological function was assigned. Most of the proteins have functions in the defense and maintenance of feline ocular surface homeostasis. Serum albumin is a bottleneck protein, with a high betweenness value. This paper is a pioneer in reporting, in-depth, the tear film proteome of domestic cats.

## 1. Introduction

Tear film (TF) is a complex and viscous trilaminar fluid composed mainly of proteins, lipids, electrolytes, and water [1], with a protective function for the ocular surface. This protection is based on the presence of proteins in the TF, which participate in the modulation of healing processes and in direct defense against pathogens [2].

Changes in the expression of tear proteins can be associated with systemic and ophthalmic diseases. Thus, its evaluation has been widely explored in human medicine as a noninvasive tool to discover molecular biomarkers for diagnosis and prognosis of several diseases [3, 4]. For the evaluation of protein expression of TF, several techniques can be used, but mass spectrometry (MS) is currently the technique of choice to identify proteins in complex biological samples such as tears [5–8].

The description of tear microcomponents is important, especially of cats, a species whose proteomic data are not well analyzed in the literature. The study of microcomponents such as proteins and their properties can be the first step for future studies of disease biomarkers in this species [1, 9].

Indeed, when compared to human medicine, in the field of veterinary medicine, little research has been published involving the TF proteome, the exceptions being studies of canines [10–12]; rabbits [13]; koalas [14]; capuchin monkeys [15]; sheep, cows, and camels [9]; and horses, three species of birds, and six species of reptiles [11]. To the best of our knowledge, no studies have been published about cats.

It is thus relevant to investigate the domestic feline TF proteome since knowledge of the proteome profile in cats' tears is the first step toward future studies of possible disease biomarkers of feline ophthalmic diseases, as well as systemic diseases. Thus, the aim of this study was to describe the proteins found in the tear film of healthy domestic cats, which was possible to be obtained through the methodology used.

## 2. Materials and Methods

### 2.1. Ethical Considerations and Animals Included in the Study

This study was approved by the Ethics Committee on Animal Experimentation Use (CEUA) of the State University of Santa Cruz (UESC) (protocols 024/15 and 03/17). All procedures were conducted in accordance with the Association for Research in Vision and Ophthalmology's (ARVO) Statement for the Use of Animals in Ophthalmic and Vision Research and the NIH Statement and also followed the Cat-Friendly Practice guidelines. The animals were donated by the owners for research after formal consent. After the experiment, all the cats were adopted.

This study included 12 mixed-breed domestic cats (*Felis catus*), of both genders, between 11 and 12 months of age, living in a controlled environment. The selected cats were previously castrated, dewormed, and immunized against the main infectious diseases. They were also certified as healthy by physical examination, had complete blood count (CBC) performed, and the concentrations of alanine aminotransferase (ALT), aspartate aminotransferase (AST), gamma glutamyl transferase (GGT), bilirubins, urea, and creatinine were within normal range. The cats were also negative for *Toxoplasma gondii*, FIV/FeLV, and feline coronavirus antibodies. In addition, a complete ophthalmic evaluation was performed, including the Schirmer tear test (STT) and intraocular pressure test, with results within the normal range, and the ocular surface was evaluated using fluorescein dyes and green lysamine (Ophthalmos, São Paulo, Brazil) and was considered healthy. All the ophthalmic tests were performed after proteomic sample collection to avoid any interference with the results.

### 2.2. Tear Film Collection

Tear film samples were collected in the morning hours, and when the tear wetted 30 mm on the Schirmer strips, the same ones used for STT-1, the strips were immediately placed in a 0.5 mL microtube (Protein LoBind Tubes; Eppendorf, São Paulo, Brazil) and placed in a thermal box until centrifugation. Immediately before centrifugation, the bottom of each microtube was punctured, and it was inserted into a larger 2.0 mL microcentrifuge tube (Protein LoBind Tubes; Eppendorf, São Paulo, Brazil) for extracting the tear fluid, as described by [16]. The TF was obtained through centrifugation (25.830 g for 10 min at 4°C) of the Schirmer strips (Ophthalmos, São Paulo, Brazil) and placed in 1.5 mL microtubes.

As tear sample amount of each animal has low volume, it was necessary to mix the individual samples of the 12 cats, place in a single 1.5 mL microtube, and keep frozen at a temperature of −20°C, until protein preparation for two-dimensional polyacrylamide gel (2D-SDS-PAGE).

### 2.3. TF Protein Quantification

The TF samples' protein was quantified by the Bradford method [17] with reading at 595 nm, using bovine serum albumin (BSA) as standard. Then, salt removal was performed to avoid influence on isoelectric focusing, thus making the sample purer. For this purpose, the total amount of 150 *μ*L of sample was mixed with 150 *μ*L of 20% TCA in plastic tubes, stored for 10 minutes on ice, and centrifuged for 5 minutes at 15,000 g. The supernatant was discarded, and the precipitate was washed twice with 200 *μ*L of 100% acetone, and once with 200 *μ*L of 80% acetone. For washes, centrifugation was done for 5 min at 18,000 g. Subsequently, the supernatant was removed, the tubes were dried at room temperature, and the contents were subsequently resuspended in 200 *μ*L of rehydration buffer (urea at 7 mol L^−1^, thiourea at 2 mol L ^−1^, 2% CHAPS, and 0.002% bromophenol blue). Then, the proteins were quantified again with the 2D Quant Kit (GE Healthcare, Illinois, USA), according to the manufacturer's instructions.

### 2.4. 2D Electrophoresis

#### 2.4.1. 1D SDS-PAGE

A volume of the sample containing 20 *μ*g of protein was mixed with a sample buffer (5% *β*-mercaptoethanol; tris-HCl, 0.02 mol L^−1^; pH, 6.8; 4% SDS; 27% glycerol; and 0.1% bromophenol blue). Then, the mixture solution and the low molecular weight marker (LMW) (14 to 97 kDa; GE Healthcare, Illinois, USA) were denatured separately at 95°C for 5 min. After this, they were both applied on the top of the 12.5% polyacrylamide gel (8 × 10 cm) in a vertical electrophoresis system (SE 260-Hoefer, San Francisco, California, USA), and LMW was inserted in the left column. The electrophoresis system was connected to a power supply in which the following running program was established: 150 V at 50 mA for 1.5 hours. After the run, the gel was placed in fixation buffer (40% alcohol and 10% acetic acid) for 1 hour, subsequently replaced by a Coomassie blue dye solution (8% sulfate ammonium, 0.8% phosphoric acid, 0.08% Coomassie Brilliant Blue G-250, and 20% methanol) for 24 hours, under agitation. Finally, the gel was conditioned with distilled water under stirring for five days to remove excess dye. After all the mentioned processes, it was possible to observe the total protein band profiles in the gel.

#### 2.4.2. 2D SDS-PAGE

A mass of 150 *μ*g of protein, in a volume of 250 *μ*L of rehydration buffer containing dithiothreitol (DTT) and 1.25 *μ*L of ampholyte, was used for isoelectric focusing. This solution was deposited on the porcelain support (strip holder), and a 13 cm dehydrated gel strip was inserted above it, with an immobilized pH gradient of 3 to 10 NL (pH 3−10NL, 130 × 3 × 0.5 mm; Immobiline ™ DryStrip, GE Healthcare, Illinois, USA), and 1 mL of DryStrip oil was added. The holder was then closed and inserted into the focuser. The protocol followed for isoelectrofocalization, in an Ettan IPGphor III system (GE Healthcare, Illinois, USA), was as follows: 12 h (overnight) at 20°C for hydration of the gel, 1 h at 500 Vh, 1 h and 4 min at 1000 Vh, 2 h and 30 min at 8000 Vh, and 22 min at 8000 Vh, in all cases at 20°C (focusing itself), totaling 17 h and 15 min.

After isoelectric focusing, the gel strips were treated for 15 min with 7 mL of equilibration buffer (urea, 6 mol L^−1^; 2% SDS; 30% glycerol; tris-HCl 0.05 mol L^−1^; pH 8.8) containing 0.21 g of 1% DTT, followed by an additional 15 minutes in 7 mL of equilibration buffer containing 175 g of 2.5% iodoacetamide. Finally, the strips were placed for 15 minutes in 7 mL of 1x running buffer (tris, 0.025 mol L^−1^; glycine, 0.19 mol L^−1^; SDS, 0.1%; pH, 8.3) [18]. In the three stages, the gel strips were kept under slow stirring. The already balanced strips were applied on top of the 12.5% polyacrylamide gel in a vertical electrophoresis system (SE 600 Ruby; GE Healthcare, Illinois, USA), and the LMW (14 to 97 kDa; GE Healthcare, Illinois, USA) was inserted on the left side on a piece of filter paper. Then, they were sealed with agarose (25 mmol L^−1^ of tris base, 192 mmol L^−1^ of glycine, 0.1% SDS, 0.5% agarose, and 0.002% bromophenol blue). The run was carried out with an electric current of 45 mA for 15 min, 120 mA for 30 min, and 150 mA, in a BioRad Power Pac 3000 source. The temperature was maintained at approximately 11°C during the entire run. At this stage, each polypeptide chain migrated according to its *m/z*, and the run was stopped when bromophenol blue reached the end of the gel.

### 2.5. Image Analysis

After the runs, each of which lasted an average of 4 hours, the gel was placed for 1 hour in fixation buffer (40% alcohol and 10% acetic acid), subsequently replaced by a Coomassie blue dye solution (8% ammonium sulfate, 0.8% phosphoric acid, 0.08% Coomassie Brilliant Blue G-250, and 20% methanol), for six days, under agitation. Then, the gel was conditioned with distilled water under stirring for five days to remove excess dye. After washing, the gel was scanned, using the LabScanner program (Amersham Bioscience, New Jersey, USA), and analyzed for spot detection, using ImageMaster 2D Platinum 7.0 software (GE Healthcare, Illinois, USA). This analysis was carried out through the combination of automatic detection of the spots by software and manual detection.

### 2.6. Protein Identification by Mass Spectrometry

The fragments of the gel were cut, using a sterile scalpel, placed in separate identified microtubes, washed for 24 hours with 200 *μ*L of 25 mmol L^−1^ NH_4_HCO_3_ in 50% ACN (pH 8) and vortexed for 10 minutes. Then, the fragments were washed with 200 *μ*L of Milli-Q water to remove the excess of the previous solution and were dehydrated in 100 *μ*L of 100% acetonitrile (ACN) for 10 min at room temperature. After this step, the supernatant was discarded, and the microtubes with the samples were dried in Concentrator 5301 (Eppendorf, São Paulo, Brazil) for approximately 10 minutes. Then, 5 *μ*L of cold trypsin gold solution (25 ng *μ*L^−1^; Promega, Madison, USA) was added, and the microtubes were kept on ice for 10 min, after which approximately 20 *μ*L of NH_4_HCO_3_ (25 mmol L^−1^) was added. The trypsin-containing spots were incubated for 16 hours in an oven at 37°C. Finally, the supernatant from the tubes was collected and transferred to new tubes, and the peptides were re-extracted with 50 *μ*L of 50% acetonitrile containing 5% formic acid, under agitation, for 30 min in a Thermo Finemixer (SH2000-DX, Singapore). The volume of each sample was reduced in the Speed Vac until it reached approximately 10–15 *μ*L.

The resulting peptides from tryptic digestion were subjected to liquid chromatography with tandem mass spectrometry (LC-MS/MS) in a nanoACQUITY system (Waters, Milford, USA) coupled to an ESI-Q-ToF micromass spectrometer (Waters, Milford, USA).

### 2.7. Interaction Network Analysis

An interaction network was built with proteins of the *Mus musculus* species homologous to the proteins identified in the proteomic analysis of the TF of cats since it is a model organism, and therefore, the network can give more information about protein interactions.

### 2.8. Statistical Analysis

The raw data were processed, and the resulting spectra were analyzed with the ProteinLynx Global SERVER 4.2 software (Waters, Milford, USA) and compared with the Swiss-Prot search (UniProtKB, https://www.uniprot.org/), using the existing database 9685-*Felis catus* (Cat) (*Felis silvestris catus*).

For the interaction network analysis, the proteins were subjected to interactome analysis using the STRING database version 11.0 (http://string-db.org). They were analyzed individually with the following parameters: (i) meaning of network edges: confidence; (ii) active interaction sources: text mining, experiments, databases, coexpression, neighborhood, gene fusion, and co-occurrence; (iii) minimum required interaction score: high confidence (0.700); (iv) maximum number of interactors to show: 1st and 2nd shell is no more than 50 interactions. The file for each network was downloaded in TSV format, and later, the files were merged and analyzed with the Cytoscape software version 3.7.1. The modularity and centrality properties (betweenness and node degree) of the network were calculated using the igraph package of the statistical tool R. For each cluster, an enrichment analysis of gene ontology was performed using the BiNGO version 3.0.3 plugin.

## 3. Results

The tear electrophoretic protein profile of cats by SDS-PAGE showed bands with molecular masses distributed in the ranges between 14 and 97 kDa, with a well-defined band at around 79 kDa, which denotes lactotransferrin (LF).

**Figure 1:**
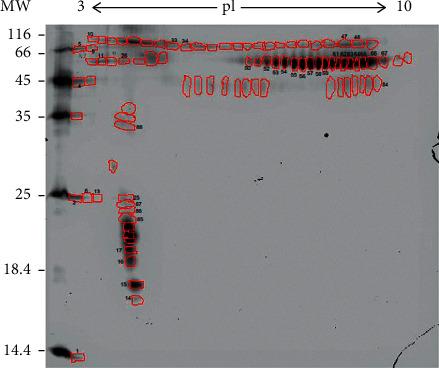
Two-dimensional polyacrylamide gel (2D-SDS-PAGE).

**Figure 2:**
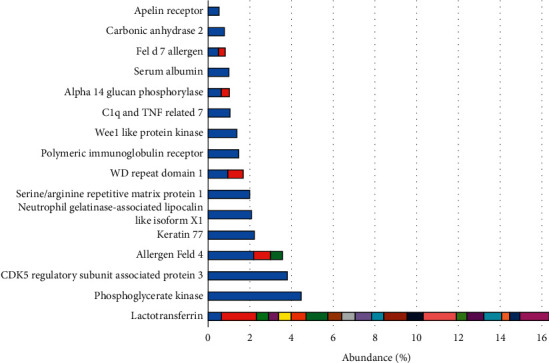
Graphical representation exposing the distribution of proteins and their abundances (%), which can be considered a signature for the protein composition of tear film of healthy cats. The different colors in the abundance bars refer to the different spots where the same protein was identified, that is, for each protein, there is a bar with the sum of all the abundances found for itself.

After processing the images, 90 spots were detected, and 40 of these were identified by mass spectrometry (Figure 1). Of these, 16 different proteins (some homologs) were identified (Figure 2). Lactotransferrin was the most abundant protein, making up approximately 16% of the TF of healthy cats, followed by phosphoglycerate kinase with approximately 5%. The protein data found in the study are described in Table 1.

**Figure 3:**
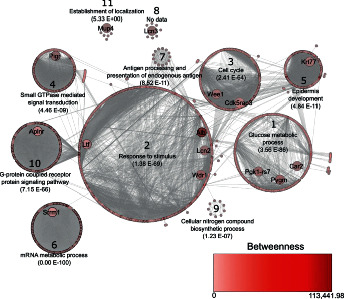
Interaction network of *Mus musculus* proteins homologous to those identified in *Felis catus*. Betweenness value represented by the color of the nodes, the lightest being the lowest value and the darkest being the highest value. It represents the ability of the node to join several clusters, or groups of nodes. Proteins with an elevated betweenness value have a high interaction with other proteins and are called bottleneck proteins. The width of the border of the nodes represents the value of node degree, so that the greater the width of the border, the greater the value and vice versa. The node degree property represents the number of connections that cross a single node, and the proteins with an above average node degree value are called hubs, which have an important regulatory function in the network. The clusters are subsets of nodes that are mostly connected to each other. In Figure 3, we have 11 clusters, each grouped in numbered circles. For each cluster, a biological process was assigned with the lowest corrected *p* value, according to the BiNGO tool. Therefore, in this network figure, for each cluster, the biological function, the number of the cluster, and its *p* value were indicated (the closer to zero, the more reliable). No annotation data were generated for cluster 8 (no data).

The interaction network of proteins homologous to those identified in *F. catus* (Figure 3) is composed of 1,000 nodes (proteins), 16,149 edges, and 11 clusters. The network contains 118 proteins considered bottlenecks (betweenness value above average), of which eight are homologous to those identified in cats' TF (Table 2). The network also contains 416 proteins considered to be hubs (above average node degree value), of which six are homologous to the proteins identified in *F. catus* (Table 2), while three of the homologous proteins identified have both characteristics—bottleneck and hub—in the network. The homologous protein of C1q and TNF-related 7 (A0A2I2V388) had confidence parameters below those established for the construction of the network.

Cluster 2 had the highest number of proteins in the network, corresponding to the stimulus response (Figure 3). Within this cluster are the homologous of lactotransferrin (spots 11, 33, 47, 48, 50, 52–59, and 61–67), serum albumin (spot 4), WD repeat domain 1 (spots 10 and 84), and neutrophil gelatinase-associated lipocalin (spot 85).

## 4. Discussion

The results of this study are pioneering in the identification of proteins present in the TF of healthy domestic cats. As already described by several researchers, knowledge of TF proteomics can assist in the investigation of ocular and systemic diseases [3, 4, 10, 11]. Thus, the results obtained here are the first step toward further studies of domestic cats, to better elucidate the behavior of numerous ocular or systemic diseases as well as their diagnosis and prognosis.

TF has been intensively explored in proteomic studies due to its easy collection and handling. However, the final volume of tears obtained from each animal depends on individual regulation of tear flow compared to other body fluids [7]. Studies directed at the proteomic evaluation of TF are increasing, both in human and veterinary medicine. Furthermore, since proteins do not act alone, but are part of a large interaction network, a systematic biology analysis was performed to obtain a broader view of the processes that take place in the TF.

After collection and processing, the final pool volume was 150 *μ*L. It was necessary to form pools to obtain an adequate final volume since TF is a type of sample that normally has small volume when compared to other samples, as reported in other studies [11, 13, 15]. Thus, analytical methods based on MS have been able to evaluate complex samples with small volume to map the protein profile of TF [14], as proven by our data.

Of the 16 proteins identified, the authors confirmed in cats what has been already found in other studies who described the presence of lactotransferrin [41], serum albumin [11, 23, 43, 44], two allergenic lipocalins [45, 46], neutrophil gelatinase-associated lipocalin (NGAL) [47], and keratin [41] in other TF studies. Three proteins (carbonic anhydrase II [22], polymeric immunoglobulin receptor [48, 49], and phosphoglycerate kinase [50]) have already been described in other structures of the lacrimal system. In addition, seven proteins (C1q/TNF-related secretory protein, three protein kinase family members, apelin receptor, *α*-1,4 glucan phosphorylase, and WD repeat domain 1) to our knowledge have never been observed in any lacrimal system structure, so they are described here for the first time.

The great majority of proteins identified have a direct link with TF functions, whether in cell development and metabolism or in the performance of the innate immune defense of the lacrimal system, functions which have already been described in the literature regarding proteins present in TF [13, 23].

The proteins carbonic anhydrase II (spot 2), *α*-1,4 glucan phosphorylase (spots 5 and 9), and phosphoglycerate kinase (spot 6) were identified. Their homologs belong to cluster 1 of the network (Figure 3), which corresponds to glucose metabolism. Carbonic anhydrase II has already been described in the lacrimal gland, the third eyelid gland, and the tarsal gland of dogs [22], but not in TF. The phosphoglycerate kinase is an important enzyme for protein biosynthesis [51], metabolic glycolysis [26], and, thus, for the metabolism of the lacrimal apparatus cells. The enzyme *α*-1,4 glucan phosphorylase catalyzes the binding of carbohydrates to proteins and lipids, forming glycoproteins. This is important for cell adhesion and signaling, as well as for protein folding [25]. To the best of our knowledge, so far, no other study has identified this peptide in the TF of any animal species.

Lactotransferrin was the peptide identified in the largest number of spots in the TF of domestic cats (20 of the 40 spots) and with the highest abundance, corresponding to approximately 16% of the cats' TF in this study. This finding corroborates the literature, which cites the presence of the protein in question in the TF of humans [44], koalas [14], and dogs [14, 52]. It is a multifunctional binding protein [29], produced by the acinar cells of the main and accessory lacrimal glands, with antimicrobial and anti-inflammatory action. Therefore, it plays an important role in innate immunity and helps maintain ocular surface homeostasis [3, 29]. Its antimicrobial action occurs because lactotransferrin inhibits the classical complement activation pathway and binds to free iron in tears, reducing the availability of iron required for bacterial growth [3, 29, 43]. This is corroborated in the interaction network since homologs of this protein have strong interaction with proteins such as myeloperoxidase (score 0.755), which is microbicidal [53] and antileukoproteinase (score 0.969), a proteinase inhibitor that modulates the immune response after a bacterial infection [54]. This explains the fact that we found lactotransferrin with high abundance in cats' TF since the samples were from healthy animals.

Serum albumin is among the proteins frequently reported in humans [23, 43, 44] and dogs' TF [11]. It has great importance in networks and, therefore, in cells and is considered a bottleneck protein with a high betweenness value, probably due to its ability to bind to various substrates such as water, Ca^2+^, Na+, K+, fatty acids, hormones, bilirubin, and drugs. In addition, it also can limit the use of iron and the growth of bacteria [55]. It is eliminated inside the tear from the conjunctival capillaries, exercising fundamental importance for the local antimicrobial defense [23] and acting as a marker of the absence of integrity of the blood-tear barrier [43, 56]. It has a strong potential to be used as a biomarker of both ophthalmic and systemic diseases since it can derive from the leakage of plasma from conjunctival vessels, mixing with TF on the ocular surface [23, 56]. A study carried out in dogs with corneal ulcers, uveitis, and glaucoma showed that the levels of tear albumin were up to 14.9 times higher in the injured eye compared to the healthy contralateral [56]. Therefore, albumin is a protein present in TF in healthy cats, and as happens in dogs, it is probably related to blood-tear barrier breakdown when present in high levels. Further studies of cats with ocular diseases must be performed to confirm this hypothesis.

NGAL, a glycoprotein member of the family of lipocalins, was also identified (spot 85), and like the allergenic lipocalins Fel d 4 and Fel d 7, it is a small extracellular protein [38, 42] capable of binding and transporting small hydrophobic molecules. In addition, lipocalins participate in immune response regulation and signal transduction, enzymatic activities [40], and cell proliferation and differentiation [38]. In veterinary medicine, when the concentration of NGAL is high [57], it has also been described as a chronic kidney injury biomarker in dogs in serum and urine [58, 59], and in cats in plasma and urine [57]. Although tears contain proteins from the lipocalin family, NGAL has only been identified in human tears [47], so there is no information about it in cats' TF, suggesting the need for further studies to investigate its applicability as an early biomarker in TF for kidney injury in cats.

WD repeat domain 1 is a cytoplasmic protein encoded by the WDR1 gene [60]. This protein's known functions are signal transduction, RNA synthesis and processing, chromatin and cytoskeleton assembly, control of the cell cycle, and participation in cell apoptosis processes [27, 28, 60]. The homologs of this protein are found within the stimulus response cluster due to their interaction with the neuronal protein proto-oncogene tyrosine-protein kinase Src (score 0.759), which participates in signaling pathways that control a diverse spectrum of biological activities including gene transcription, immune response, cell adhesion, cell cycle progression, and apoptosis. Thus, the action of WD repeat domain 1 on the feline lacrimal system may be associated with the growth and structure of the cells that compose it. There are no reports in the literature of the presence of this protein in TF.

Homologs of cyclin-dependent protein kinase 5 (CDK5) associated with protein 3 (CDK5RAP3; spot 25) and Wee1-like protein kinase (spot 88) are part of cluster 3, corresponding to the cell cycle. CDK5RAP3 is a unit regulator and linker of protein kinase, responsible for controlling the cell cycle from growth, differentiation to cell apoptosis [33] and can assist in the healing of corneal lesions, by stimulating the adhesion of corneal epithelial cells to one another when facing an injury [61]. In turn, Wee1 belongs to the serine/threonine kinase family and is responsible for regulating the G2 phase of the cell cycle, preventing the mitosis of cells that have undergone mutation, thus preventing DNA replication [42]. Thus, we suggest that such proteins found in cats' TF participate in the control of the development, maturation, and proliferation of the cells of the corneal epithelial layer.

Still, in this context, serine/arginine repetitive matrix protein 1 (SRRM1) (spot 34) also belongs to the serine-threonine kinase family [62]. Its homolog belongs to cluster 6, which represents the metabolic process of mRNA, since it is a protein encoded by the SRRM1 gene, hence also known by this acronym, and participates in several stages of mRNA processing and maturation, such as splicing [35–37]. Both CDK5RAP3 and SRRM1, reported in the TF of cats, have not yet been identified in any other species studied so far. In this regard, since the mRNA metabolism is essential for protein synthesis, this explains the presence of these components, active in the processing and maturation of the mRNA, in healthy cats' TF.

The polymeric immunoglobulin receptor (spot 26) and apelin receptor (spot 1) were identified. The homolog of the first one is part of cluster 4, and the second one, of cluster 10. Both correspond to processes related to signal transduction. The polymeric immunoglobulin receptor consists of a transmembrane domain receptor [48] that binds to IgA and IgM immunoglobulins [34], and IgA is abundantly present in TF. It is important for the innate mucosal immune response [63], and this has already been reported in other studies [48, 49], but not in TF samples of any animal species so far.

On the other hand, the apelin receptor (spot 1) is a peptide that activates and stimulates apelin function after binding. It is expressed in diverse cells and tissues, such as heart, blood vessels, and adipocytes [19, 20]. Apelin belongs to the family of adipokines, which are part of a group of hormones and cytokines with proinflammatory action [64]. To our knowledge, this protein has not been described in TF of any species until now. Tracing an extension to its function in the ocular system, we suggest it is important for the formation of the vessels that nourish this organ, as well as possibly being present in the lipid component of TF.

The allergenic lipocalins Fel d 7 (spots 14 and 15) and Fel d 4 (spots 16, 17 and 86) are part of the family of lipocalins that are often reported in the TF of humans as proteins that bind to lipids and other proteins [45, 46]. This specific class of allergenic lipocalins has only been previously described in dogs' TF, as one of the most abundant proteins [11]. Fel d 7 and Fel d 4 are found in body fluids and secretions, such as tears [31, 32, 65]. Thus, the study of these lipocalins can assist in the diagnosis of allergic reactions to cats.

Additionally, the protein related to C1q/TNF was also characterized, considered a secretory protein that belongs to the same family as adiponectin, both secreted by adipose tissue [30]. C1q/TNF 1 to 10 are part of this family, and although the metabolic functions of adiponectin are well characterized [66], the physiological processes regulated by C1q/TNF need to be better explored. It is known that C1q/TNF 3 influences energy metabolism and insulin sensitivity [67] possibly secreted in the lipid layer of the TF.

Finally, keratin was also found on the TF of cats probably came from the epithelial and myoepithelial cells present in the lacrimal and meibomian glands, with the purpose of interacting with the TF lipid layer [68].

## 5. Conclusions

Most of the proteins identified have important functions in the defense and maintenance of homeostasis of the feline ocular surface. The relative abundance of all proteins found in this study promotes the first dispersion pattern, which can be considered a signature of the behavior and distribution of proteins in healthy cats.

The findings of the interaction network explain why lactotransferrin was present as the most abundant protein, because the samples were from healthy cats. The homologs of this protein showed high interaction with myeloperoxidase and antileukoproteinase, playing an important role in innate immunity and maintenance of ocular surface homeostasis. Serum albumin was equally identified, and the network demonstrated through the high betweenness value that this protein behaves as a bottleneck protein in cats' TF, probably due to its ability to bind to various substrates.

Seven proteins are described here for the first time in TF, cyclin-dependent protein kinase, serine/arginine repetitive matrix protein, apelin receptor, secretory protein related to C1q/TNF, Wee1, *α*-1,4 glucan phosphorylase, and WD repeat domain 1. Currently, there are more advanced techniques for proteomic analysis of biological samples, which can generate more accurate and complete results regarding the protein composition of the tear film, but at the moment, this equipment was the one available to our team. Despite this, this study brings the first description of proteins in cats' TF and is the first step towards future studies about biomarkers of feline ophthalmic and systemic diseases. Thus, from this study, we obtained a first step of knowledge of the main proteins present in the tear film of healthy domestic cats and must stimulate new studies covering a larger number of samples and employing more advanced techniques.

## Figures and Tables

**Table 1:** Peptides identified in the tear of healthy cats.

Spot	Protein	Access NCBI/UniProt	Gene	Isoelectric point	Probability (%)	Recognized sequence	Molecular function
1	Apelin receptor	M3X9N6_FELCA	APLNR	7.7913	99.32	2	It modifies cellular activity when it binds to the apelin [19, 20]
2	Carbonic anhydrase 2	M3W3J4_FELCA	CA2	7.0276	100	9	Reversible catalysis of hydration of bicarbonate and dehydration of carbonic acid [21, 22]
4	Serum albumin	A0A2I2U7Y0_FELCA	ALB	5.1687	83.14	5	Antimicrobial defense and healing [23, 24]
5	*α*-1, 4 glucan phosphorylase	A0A337SC59_FELCA	PYGM	8.5646	62.24	33	It catalyzes the formation of glycoprotein [25]
6	Phosphoglycerate kinase	A0A337SHN8_FELCA	PGK1	7.9065	79.49	5	Interaction with ATP [26]
9	*α*-1, 4 glucan phosphorylase	A0A337SC59_FELCA	PYGM	8.5646	62.31	27	It catalyzes the formation of glycoprotein [25]
10	WD repeat domain 1	M3WEW2_FELCA	WDR1	6.1917	81.97	5	Actin ligand [27, 28]
11	Lactotransferrin	M3WKU0_FELCA	LTF	7.6575	59.97	24	Binding protein, multifunctional [29]
13	C1q and TNF-related 7	A0A2I2V388_FELCA	C1QTNF7	5.1962	85.26	2	Adiponectin paralogs [30]
14	Fel d 7 allergen	E5D2Z5_FELCA	LOC100533977	4.6666	94.62	15	Binding to small hydrophobic molecules and IgE [31, 32]
15	Fel d 7 allergen	E5D2Z5_FELCA	LOC100533977	4.6666	80.18	15	Binding to small hydrophobic molecules and IgE [31, 32]
16	Allergen Fel d 4	ALL4_FELCA	Not described	4.7216	99.99	7	Binding to small hydrophobic molecules [31, 32]
17	Allergen Fel d 4	ALL4_FELCA	Not described	4.7216	99.99	5	Binding to small hydrophobic molecules and IgE [31, 32]
25	CDK5 regulatory subunit-associated protein 3	A0A337SKT3_FELCA	CDK5RAP3	4.717	42.32	3	Binding to protein kinase [33]
26	Polymeric immunoglobulin receptor	M3W5Z6_FELCA	PIGR	5.4053	100	10	Binding to IgA and IgM [34]
33	Lactotransferrin	M3WKU0_FELCA	LTF	7.6575	86.26	22	Multifunctional binding protein [29]
34	Serine/arginine repetitive matrix protein 1 isoform X2 (SRRM1)	XP_030879145.1	TRY50791.1	9.9902	35.85	3	Participates in several stages of mRNA processing and maturation, such as splicing [35–37]
47	Lactotransferrin	M3WKU0_FELCA	LTF	7.6575	54.38	20	Multifunctional binding protein [29]
48	Lactotransferrin	M3WKU0_FELCA	LTF	7.6575	50.57	21	Multifunctional binding protein [29]
50	Lactotransferrin	M3WKU0_FELCA	LTF	7.6575	50.57	17	Multifunctional binding protein [29]
52	Lactotransferrin	A0A2I2V3V6_FELCA	LTF	7.7844	50.22	29	Multifunctional binding protein [29]
53	Lactotransferrin	M3WKU0_FELCA	LTF	7.6575	72.83	30	Multifunctional binding protein [29]
54	Lactotransferrin	M3WKU0_FELCA	LTF	7.6575	72.01	30	Multifunctional binding protein [29]
55	Lactotransferrin	M3WKU0_FELCA	LTF	7.6575	100	44	Multifunctional binding protein [29]
56	Lactotransferrin	M3WKU0_FELCA	LTF	7.6575	100	41	Multifunctional binding protein [29]
57	Lactotransferrin	M3WKU0_FELCA	LTF	7.6575	100	37	Multifunctional binding protein [29]
58	Lactotransferrin	M3WKU0_FELCA	LTF	7.6575	59.77	35	Multifunctional binding protein [29]
59	Lactotransferrin	M3WKU0_FELCA	LTF	7.6575	81.42	33	Multifunctional binding protein [29]
61	Lactotransferrin	M3WKU0_FELCA	LTF	7.6575	98.63	40	Multifunctional binding protein [29]
62	Lactotransferrin	M3WKU0_FELCA	LTF	7.6575	100	36	Multifunctional binding protein [29]
63	Lactotransferrin	M3WKU0_FELCA	LTF	7.6575	100	38	Multifunctional binding protein [29]
64	Lactotransferrin	M3WKU0_FELCA	LTF	7.6575	100	40	Multifunctional binding protein [29]
65	Lactotransferrin	M3WKU0_FELCA	LTF	7.6575	100	42	Multifunctional binding protein [29]
66	Lactotransferrin	M3WKU0_FELCA	LTF	7.6575	100	45	Multifunctional binding protein [29]
67	Lactotransferrin	M3WKU0_FELCA	LTF	7.6575	50.04	33	Multifunctional binding protein [29]
84	WD repeat domain 1	M3WEW2_FELCA	WDR1	6.1917	91.53	5	Actin ligand [27, 28]
85	Neutrophil gelatinase-associated lipocalin-like isoform X1 (NGAL)	XP_003995976.1	LOC101098498	5.0149	62.29	7	Binding and transporting small hydrophobic molecules in addition to participation in immune responses, signal transfunction, and cell differentiation [38–40]
86	Allergen Fel d 4	ALL4_FELCA	Not described	4.7216	78.38	8	Binding to small hydrophobic molecules and IgE [31, 32]
87	Keratin 77	M3XG51_FELCA	KRT77	6.8177	100	4	Structural protein present in epithelial cells [41]
88	Wee1-like protein kinase	M3X956_FELCA	WEE1	6.1725	67.81	3	Regulation of DNA replication [42]

**Table 2:** *Mus musculus* proteins homologous to the proteins identified in the tear film of cats (*Felis catus*).

Uniprot/NCBI ID (*F. catus*)^a^	Nome (*Felis catus*)^b^	Homóloga (*M. musculus*)^c^	Identity (%)^d^	BN^e^	Hub^f^	Cluster^g^
E5D2Z5	Fel d 7 allergen	Lcn3	39	N	N	8
ALL4	Allergen Fel d 4	Mup4	60	N	N	11
M3W5Z6	Polymeric immunoglobulin receptor	Pigr	64	Y	Y	4
XP_003995976.1	Neutrophil gelatinase-associated lipocalin-like isoform X1 (NGAL)	Lcn2	65	Y	N	2
M3WKU0	Lactotransferrin	Ltf	67	Y	Y	2
A0A2I2U7Y0	Serum albumin	Alb	73	Y	Y	2
M3W3J4	Carbonic anhydrase 2	Car2	82	Y	N	1
A0A337SKT3	CDK5 regulatory subunit-associated protein 3	Cdk5rap3	85	N	N	3
M3X956	Wee1-like protein kinase	Wee1	86	N	N	3
A0A337SC59	*α*-1, 4 glucan phosphorylase	Pygm	87	Y	N	1
M3X9N6	Apelin receptor	Aplnr	88	N	Y	10
A0A337SHN8	Phosphoglycerate kinase	Pgk1-rs7	96	Y	N	1
XP_030879145.1	Serine/arginine repetitive matrix protein 1 isoform X2 (SRRM1)	Srrm1	99	N	Y	6
M3XG51	Keratin 77	Krt77	99	N	Y	5
M3WEW2	WD repeat domain 1	Wdr1	101	Y	N	2

## Data Availability

The data used to support the findings of this study are included within the article.
